# The Pasteur Institute

**Published:** 1889-04

**Authors:** 


### THE PASTEUR INSTITUTE.

The great biological institute established in Paris by public subscription for carrying forward the work of M. Pasteur opened, by the President of the French Republic, assisted by other French and foreign dignitaries, is perhaps the most extraordinary recognition ever received by a scientific discoverer. The subscriptions for building and endowment amounted to over half a million dollars. The French Government has allotted $25,000 a year to sustain the current expenses; all the anti-rabic treatment of patients being gratuitous, except that those who are able may contribute to the funds.

Human beings were first inoculated with Pasteur's attenuated virus in 1885. Up to July 1, 1888, 5,384 persons had been treated in Paris. In 1886 the number inoculated was 2,681, in 1887 it was 1,778, and in the first six months of the present year it was 914. The rate of mortality in the first year was 1.34 percent. It fell to 1.12 in the second year, and in the first half of the present year it was only .77. The deaths of persons in whom the poison had become developed when they were subjected to treatment. It has been estimated that before Pasteur's method was used the mortality was about 16 per cent. of the persons bitten.

There have been established 17 branch laboratories. Seven of these are in Russia, five are in Italy, and each of five other countries—Roumania, Austria, Brazil, Cuba, and the Argentine Republic—has one. Nearly 4,000 cases have been treated in these branch institutions. At St. Petersburg 484 have been inoculated, with a mortality of 2.68 per cent. The number of those treated at Odessa has been 1,135, the rate of mortality under the mild treatment in 1886 having been 3.39, while in the two following years, under the "intensive" treatment, it fell to .58 and .65 respectively. In Havana 170 were inoculated, with a mortality rate of .6 per cent. The high rate of mortality in Russia is accounted for by the fact that many of the patients had been bitten by mad volves.

The Institute is only for the protective inoculation of persons bitten by rabid animals, but also for the prosecution of bacteriological researches in general, such as those of Dr. Gamaleia, who is pursuing the microbe of Asiatic cholera, and of Dr. Gibier, who is seeking the microbe of yellow fever.

